# [^18^F]Fluspidine—A PET Tracer for Imaging of σ_1_ Receptors in the Central Nervous System

**DOI:** 10.3390/ph17020166

**Published:** 2024-01-28

**Authors:** Friedrich-Alexander Ludwig, Erik Laurini, Judith Schmidt, Sabrina Pricl, Winnie Deuther-Conrad, Bernhard Wünsch

**Affiliations:** 1 Institute of Radiopharmaceutical Cancer Research, Department of Neuroradiopharmaceuticals, Helmholtz-Zentrum Dresden-Rossendorf, D-04318 Leipzig, Germany; f.ludwig@hzdr.de (F.-A.L.); w.deuther-conrad@hzdr.de (W.D.-C.); 2 Molecular Biology and Nanotechnology Laboratory (MolBNL@UniTS), DEA, University of Trieste, 34127 Trieste, Italy; erik.laurini@dia.units.it (E.L.); sabrina.pricl@dia.units.it (S.P.); 3 Institut für Pharmazeutische und Medizinische Chemie, Universität Münster, Corrensstraße 48, D-48149 Münster, Germany; judith.schmidt7@uni-muenster.de; 4 Department of General Biophysics, Faculty of Biology and Environmental Protection, University of Lodz, 90-136 Lodz, Poland; 5 GRK 2515, Chemical Biology of Ion Channels (Chembion), Universität Münster, Corrensstraße 48, D-48149 Münster, Germany

**Keywords:** σ_1_ receptor ligands, σ_1_:σ_2_ selectivity, structure–affinity relationships, enantioselective synthesis, pharmacokinetics, logD_7.4_ value, plasma protein binding, biotransformation, molecular dynamics simulations, ligand–σ_1_ receptor interactions, radiosynthesis, automated radiosynthesis, radiometabolites, biodistribution, irreversible binding of (*R*)-[^18^F]**2**, major depressive disorder, increased σ_1_ receptor density, occupancy study with pridopidine

## Abstract

σ_1_ receptors play a crucial role in various neurological and neurodegenerative diseases including pain, psychosis, Alzheimer’s disease, and depression. Spirocyclic piperidines represent a promising class of potent σ_1_ receptor ligands. The relationship between structural modifications and σ_1_ receptor affinity and selectivity over σ_2_ receptors led to the 2-fluoroethyl derivative fluspidine (**2**, *K*_i_ = 0.59 nM). Enantiomerically pure (*S*)-configured fluspidine ((*S*)-**2**) was prepared by the enantioselective reduction of the α,β-unsaturated ester **23** with NaBH_4_ and the enantiomerically pure co-catalyst (*S*,*S*)-**24**. The pharmacokinetic properties of both fluspidine enantiomers (*R*)-**2** and (*S*)-**2** were analyzed in vitro. Molecular dynamics simulations revealed very similar interactions of both fluspidine enantiomers with the σ_1_ receptor protein, with a strong ionic interaction between the protonated amino moiety of the piperidine ring and the COO^-^ moiety of glutamate 172. The ^18^F-labeled radiotracers (*S*)-[^18^F]**2** and (*R*)-[^18^F]**2** were synthesized in automated syntheses using a TRACERlab FX FN synthesis module. High radiochemical yields and radiochemical purity were achieved. Radiometabolites were not found in the brains of mice, piglets, and rhesus monkeys. While both enantiomers revealed similar initial brain uptake, the slow washout of (*R*)-[^18^F]**2** indicated a kind of irreversible binding. In the first clinical trial, (*S*)-[^18^F]**2** was used to visualize σ_1_ receptors in the brains of patients with major depressive disorder (MDD). This study revealed an increased density of σ_1_ receptors in cortico-striato-(para)limbic brain regions of MDD patients. The increased density of σ_1_ receptors correlated with the severity of the depressive symptoms. In an occupancy study with the PET tracer (*S*)-[^18^F]**2**, the selective binding of pridopidine at σ_1_ receptors in the brain of healthy volunteers and HD patients was shown.

## 1. Introduction: The Role of σ_1_ Receptors in Some Brain Diseases

The σ_1_ receptor is highly expressed in the central nervous system (CNS) and therefore is centrally involved in various pathological conditions of the CNS. Therefore, the labeling of σ_1_ receptors with a positron emission tomography (PET) tracer, allowing non-invasive imaging, represents a valuable tool for target validation as well as diagnosis and prognosis. In this report, we will focus on the participation of the σ_1_ receptor in pain, psychosis, Alzheimer’s disease, and depression [1,2,3].

### 1.1. Pain

**Figure 1:**
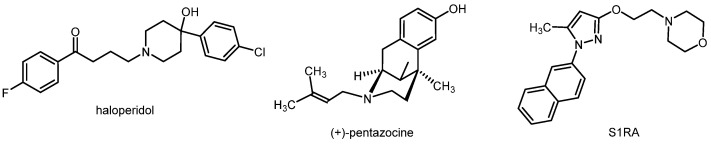
σ_1_ receptor ligands modulating pain perception. The σ_1_ receptor antagonist S1RA is active in the treatment of neuropathic pain.

A functional connection between the σ_1_ and opioid receptor systems has been reported. The downregulation of the σ_1_ receptor led to an increased analgesic activity of opioid analgesics [2]. The same effect could be produced by σ_1_ receptor antagonists such as haloperidol (Figure 1). On the other hand, σ_1_ receptor agonists (e.g., (+)-pentazocine, Figure 1) resulted in reduced analgesia mediated by opioid (MOR) agonists [4]. However, σ_1_ receptors are also directly involved in pain sensation, in particular in the special type of neuropathic pain. While capsaicin produced mechanical allodynia in wild-type mice, it failed to induce the same effect in σ_1_ receptor knock-out mice. Furthermore, σ_1_ receptor antagonists could dose-dependently reduce capsaicin-mediated mechanical allodynia, while σ_1_ receptor agonists reversed this analgesic effect [5]. The σ_1_ receptor antagonist S1RA (Figure 1) represents the most advanced drug candidate, exhibiting high analgesic activity in animal models of neuropathic pain. S1RA is currently being investigated in a phase II clinical trial for the treatment of neuropathic pain [6,7].

### 1.2. Psychosis

**Figure 2:**
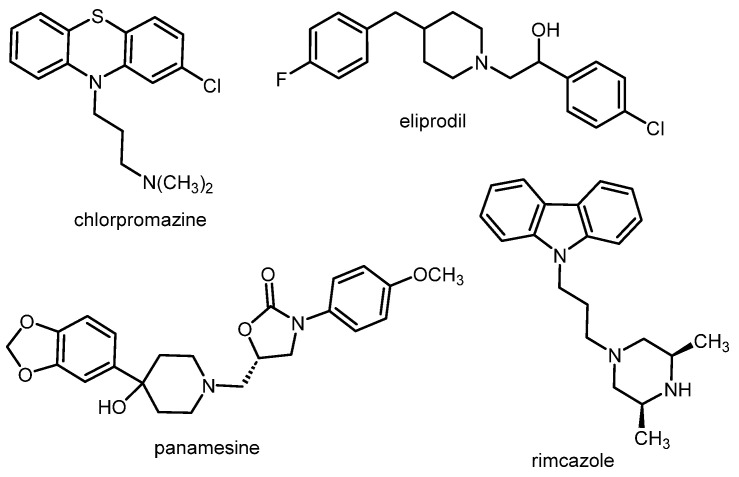
σ_1_ receptor antagonists with antipsychotic potential.

Classical antipsychotics inhibit the G protein-coupled dopamine D_2_ receptor. However, the prototypical antipsychotic haloperidol (Figure 1) inhibits not only D_2_ receptors (binding affinity: *K*_i_(D_2_) = 2.6 nM) [8], but also σ_1_ receptors with high affinity (*K*_i_(σ_1_) = 6.2 nM) [9]. Other clinically used antipsychotics (e.g., chlorpromazine as a member of the class of phenothiazines, Figure 2) also show moderate to high σ_1_ receptor affinity [10]. It was hypothesized that the inhibition of σ_1_ receptors is capable of contributing to the overall antipsychotic effects of these D_2_ receptor antagonists [11]. In addition to these “mixed D_2_/σ_1_ receptor antagonists”, some ligands predominantly inhibiting σ_1_ receptors (e.g., eliprodil (SL82.0715), rimcazole (BW234U), panamesine (EMD57445), DuP734, and BMY14802 (BMS181100)) were tested in clinical trials for their potential use as antipsychotics. In Figure 2, the structures of eliprodil, rimcazole, and panamesine are shown, exemplarily [12]. 

### 1.3. Alzheimer’s Disease

**Figure 3:**
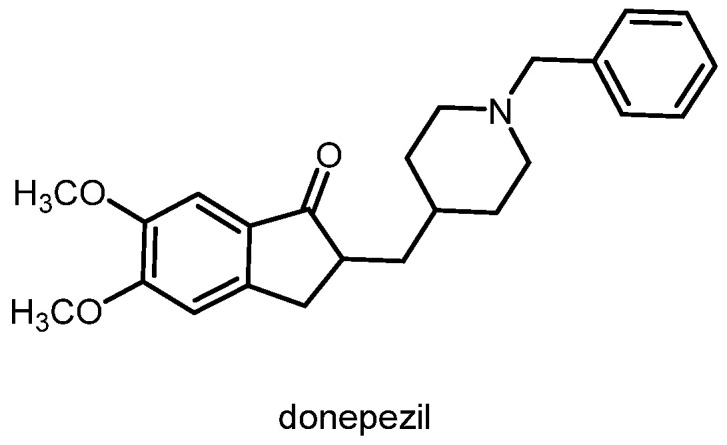
Donepezil, an acetylcholinesterase inhibitor and σ_1_ receptor agonist used for the treatment of Alzheimer’s disease.

The σ_1_ receptor agonist (+)-pentazocine was able to attenuate the memory deficits in mice treated with amyloid β_25–35_ (cerebroventricular application) in a dose-dependent mode. In animal models of Alzheimer’s disease (chronic intracerebroventricular infusion of the amyloid β_1-40_ protein), σ_1_ receptor agonists improved depressive behavior in mice [13,14]. The acetylcholinesterase inhibitor donepezil (Figure 3) is one of the first-line drugs used for the treatment of Alzheimer’s disease. In addition to the inhibition of acetylcholinesterase, donepezil can also activate σ_1_ receptors. It was hypothesized that the activation of σ_1_ receptors by donepezil contributes to its neuroprotective and anti-amnesic effects [15]. In PET studies, a low σ_1_ receptor density was found for patients at an early stage of Alzheimer’s disease [16].

### 1.4. Depression

**Figure 4:**
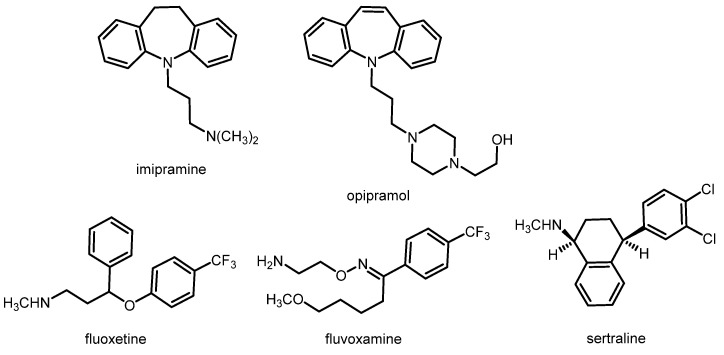
Clinically used antidepressants with σ_1_ receptor agonistic activity.

Various studies have shown that σ_1_ receptors are involved in the pathology of depression. A depressive-like behavior was observed in σ_1_ receptor knock-out mice [17]. In the forced swimming test, which is used to evaluate antidepressant drugs, some σ_1_ receptor agonists exhibited antidepressant activity [18,19]. Furthermore, several clinically used antidepressants, such as tricyclic antidepressants and selective serotonin reuptake inhibitors (SSRIs), show moderate to high σ_1_ receptor affinity. Figure 4 displays imipramine and opipramol as examples of the class of tricyclic antidepressants, and fluvoxamine, fluoxetine, and sertraline as examples of SSRIs. The repeated treatment of rats with the antidepressants imipramine and fluvoxamine, but also with the σ_1_ receptor agonist (+)-pentazocine, led to the downregulation of σ_1_ receptors in some regions of the brain, including the striatum, hippocampus, and cerebral cortex [20,21]. 

## 2. Fluorinated PET Tracers Derived from the Promising σ_1_ Ligand Cutamesine (SA 4503)

The introduction of fluorine-18 into an aromatic ring requires the careful selection of appropriate precursors and longer reaction sequences. The introduction of fluorine-18 into aliphatic side chains by S_N_2-substitution is straightforward. However, aryl fluorides are usually rather stable in vivo, while alkyl fluorides carry the risk of defluorination, which would result in bone labeling via the formation of [^18^F]CaF_2_. Several PET tracers bearing aromatic and aliphatic fluorine-18 have been reported in the literature [16,22,23]. 

**Figure 5:**
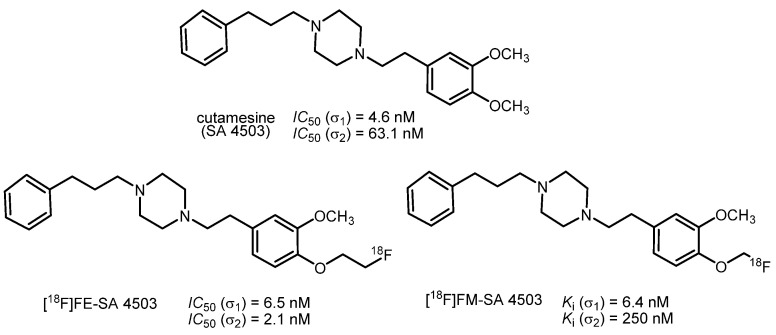
σ_1_ Receptor agonist cutamesine (SA 4503) and derived 18-F-labeled PET tracers [^18^F]FE-SA 4503 and [^18^F]FM-SA 4503.

The most prominent fluorinated σ_1_ receptor-targeting PET tracers are derived from the σ_1_ receptor agonist cutamesine (SA 4503, Figure 5) [24]. Cutamesine has anti-amnestic activity and can reduce amnesia caused by REM sleep deprivation [25,26]. Furthermore, this σ_1_ receptor agonist showed antidepressant activity in the forced swimming test of rodents [27].

The fluoroethyl derivative FE-SA 4503 showed only moderate to low selectivity for the σ_1_ receptor over the σ_2_ subtype. In a PET study with rhesus monkeys, [^18^F]FE-SA 4503 exhibited fast uptake in the brain and enrichment in regions with high expression of σ_1_ receptors. However, an equilibrium of ligand binding to the σ_1_ receptor was not reached within 90 min [28]. Despite its α-fluoro ether substructure, the fluoromethyl derivative FM-SA 4503 displayed an unexpectedly high chemical stability in vitro and in vivo. The replacement of [^18^F]FM-SA 4503 with haloperidol in a study with rhesus monkeys revealed a higher specific binding of [^18^F]FM-SA 4503 than of [^11^C]SA 4503. Although [^18^F]FM-SA 4503 has an unconventional structure, it represents a promising fluorinated PET tracer for the labeling of σ_1_ receptors in the brain [29].

The ^18^F-labeled PET-tracers [^18^F]FE-SA 4503 and [^18^F]FM-SA 4503 (Figure 5) were synthesized in two-step sequences. At first, the ^18^F-labeled reagents [^18^F]FCH_2_CH_2_OTs and [^18^F]CH_2_BrF were prepared by the nucleophilic substitution of TsOCH_2_CH_2_OTs and H_2_CBr_2,_ respectively, with the K[^18^F]F/Kryptofix system. In the second step, the phenolate precursor was reacted with [^18^F]FCH_2_CH_2_OTs and [^18^F]H_2_CBrF to afford the ^18^F-labeled PET tracers [^18^F]FE SA4503 and [^18^F]FM SA4503, respectively [28,29].

## 3. Spirocyclic σ_1_ Receptor Ligands Designed for PET Studies: Structure Affinity Relationships

We are particularly interested in drugs with a large number of sp^3^-hybridzed C-atoms incorporated in rigid scaffolds, such as spirocyclic, bicyclic, or propellane systems leading to defined three-dimensional frameworks with an exact orientation of functional groups and substituents. Therefore, we started to exploit the pharmacological potential, in particular the σ_1_ receptor affinity and selectivity, of spirocyclic piperidines.

A homologous series of fluoroalkyl-substituted spirocyclic piperidines **1**–**4** bearing a benzyl moiety at the piperidine N-atom was prepared. The four homologs **1**–**4** showed very high σ_1_ receptor affinities (*K*_i_ = 0.59–1.4 nM) and low σ_2_ receptor affinity (*K*_i_ = 489–837 nM), resulting in a high σ_1_:σ_2_ selectivity [30,31,32,33,34]. The highest σ_1_ receptor affinity and σ_1_:σ_2_ selectivity were observed for the fluoroethyl derivative **2** (Table 1). Therefore, the benzyl moiety of **2** was replaced by several other substituents including substituted benzyl, cyclohexylmethyl, and alkyl moieties. With the exception of the very lipophilic *n*-butyl and *n*-octyl derivatives **8** and **9**, the modified spirocyclic piperidines **5**–**7** and **10** displayed high σ_1_ receptor affinity, but reduced selectivity over the σ_2_ receptor. In particular, the cyclohexylmethyl derivative **6** binds with subnanomolar affinity at σ_1_ receptors (K_i_ = 0.71 nM), but also with moderate affinity at σ_2_ receptors (K_i_ = 57 nM), leading to a low σ_1_:σ_2_ selectivity [31]. The very high σ_1_ receptor affinity (K_i_ = 0.59 nM) and σ_1_:σ_2_ selectivity (1331-fold) and the promising physicochemical properties (see Chapter 5) prompted us to further develop the fluoroethyl derivative **2**, which was termed fluspidine.

## 4. Synthesis of Racemic and Enantiomerically Pure Fluspidine (2, (*S*)-2 and (*R*)-2)

**Scheme 1:**
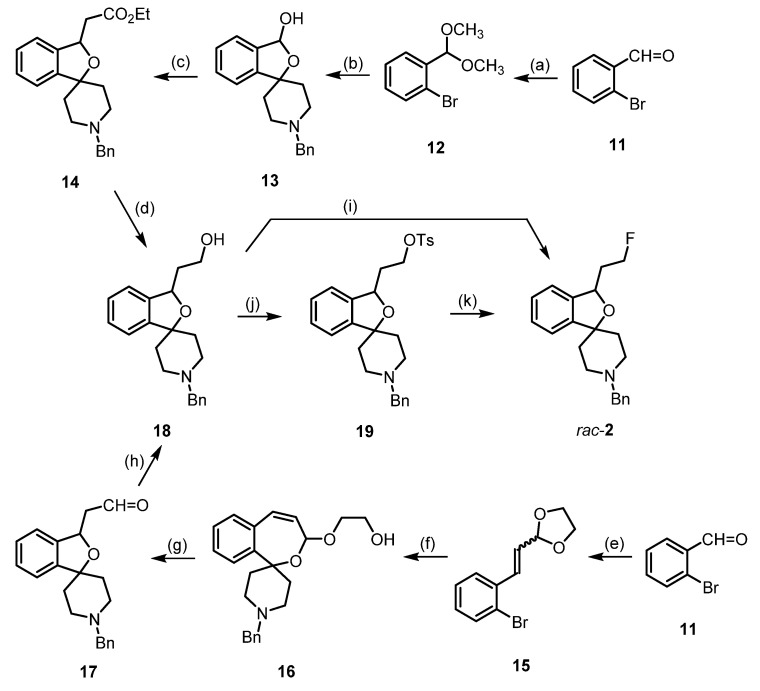
Synthesis of racemic fluspidine *rac*-**2**. Reagents and reaction conditions: (a) CH_3_OH. HC(OCH_3_)_3_, TsOH, reflux, 85%. (b) 1. *n*-BuLi, −95 °C; 2. 1-benzylpiperidin-4-one, −95 °C, then rt; 3. HCl, THF, 66 °C, 11%. (c) Ph_3_P=CHCO_2_Et, Cs_2_CO_3_, toluene, reflux, 88%. (d) LiAlH_4_, Et_2_O, −15 °C, 75%. (e) [(1,3-dioxolan-2-yl)methyl]triphenylphosphonium bromide [(CH_2_O)_2_CHCH_2_PPh_3_Br], K_2_CO_3_, TDA-1 [tris(methoxyethoxyethyl)amine, 95%; (f) *n*-BuLi, −78 °C, then addition of 1-benzylpiperidin-4-one, −78 °C, then rt, 67%. (g) HCl, 2 M, 37%. (h) NaBH_4_, CH_3_CN, 37%. (i) DAST, CH_2_Cl_2_, −78 °C, then rt, 56%. (j) TsCl, DMAP, NEt_3_, CH_2_Cl_2_, 17 h, rt, 36%. (k) Bu_4_NF, THF, 4 h, rt, 80%.

For the synthesis of racemic fluspidine (**2**), two synthetic routes were pursued, both of which started with 2-bromobenzaldehyde (**11**) (Scheme 1). After the conversion of aldehyde **11** into dimethyl acetal **12**, bromine–lithium exchange with *n*-BuLi led to an aryllithium intermediate, which reacted with 1-benzylpiperidin-4-one to provide the cyclic hemiacetal **13** after hydrolysis with HCl. The Domino reaction of the hemiacetal **13** with the Wittig reagent Ph_3_P=CHCO_2_Et consisted in the opening of the hemiacetal to form an hydroxy aldehyde, the Wittig reaction of the aldehyde to give an α,β-unsaturated ester, and finally an intramolecular Michael addition of the tertiary alcohol to the α,β-unsaturated ester to end up with the spirocyclic ester **14**. The LiAlH_4_ reduction of the ester **14** provided the primary alcohol **18** [35].

According to the second route, the Wittig reaction of aldehyde **11** was first performed, leading to the α,β-unsaturated acetal **15**. Bromine-lithium exchange followed by reaction with 1-benzylpiperidin-4-one provided the spirocyclic 2-benzoxepine **16**, which upon hydrolysis with HCl led to ring contraction, giving the (2-benzofuranyl)acetaldehyde **17**. Aldehyde **17** was reduced with NaBH_4_ to give the primary alcohol **18** [31] (Scheme 1).

The fluorinating reagent DAST (diethylaminosulfur trifluoride, Et_2_NSF_3_) converted the intermediate primary alcohol **18** directly into the fluoroethyl derivative **2** [31]. To prepare the radiosynthesis, the primary alcohol **18** was transformed into the tosylate **19**, which reacted with TBAF (tetrabutylammonium fluoride, Bu_4_N^+^F^−^) to produce fluspidine (**2**) [36] (Scheme 1).

**Scheme 2:**
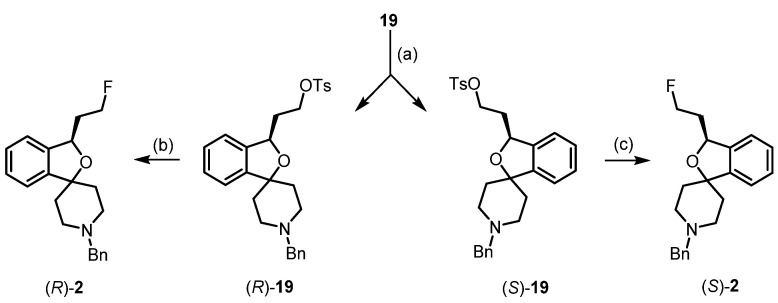
Synthesis of enantiomerically pure fluspidine enantiomers (*R*)-**2** and (*S*)-**2** by chiral resolution of racemic tosylate **19**. Reagents and reaction conditions: (a) Daicel^®^ Chiralpak IB, 5 µm, 250 mm/20 mm. Eluent, isohexane:ethanol, 100:2; flow rate, 10.0 mL/min. (*R*)-**19**: t_R_ = 26–27 min, 98.2%ee. (*S*)-**19**: t_R_ = 28–36 min. 97.8%ee. (b) Bu_4_NF, THF, rt, 3.5 h, 69%, 99.6%ee. (c) Bu_4_NF, THF, rt, 17 h, 80%, 96.4%ee.

In order to obtain pure fluspidine enantiomers (*R*)-**2** and (*S*)-**2**, the chiral resolution of racemic tosylate **19** was conducted. The enantiomeric tosylates (*R*)-**19** and (*S*)-**19** were separated using the Daicel^®^ Chiralpak IB column and the eluent isohexane:ethanol 100:2, and the absolute configuration of the enantiomers was determined by CD spectroscopy. Both enantiomers, (*R*)-**19** and (*S*)-**19**, were converted into the fluspidine enantiomers (*R*)-**2** (99.6%ee) and (*S*)-**2** (96.4%ee) using TBAF (Bu_4_NF) as a fluoride source for the S_N_2 substitution [36] (Scheme 2).

**Scheme 3:**
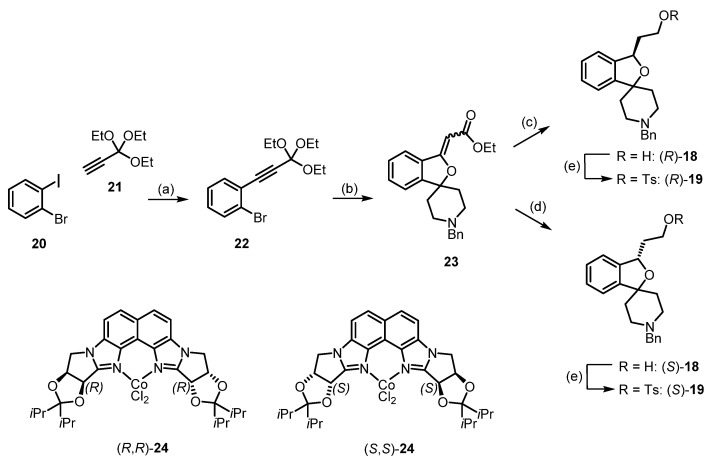
Synthesis of enantiomerically pure alcohols (*R*)-**18** and (*S*)-**18** by enantioselective reduction of α,β-unsaturated ester **23**. Reagents and reaction conditions: (a) Sonogashira coupling—PdCl_2_(PPh_3_)_2_, CuI, NEt_3_, THF, rt, 15 h, 47%. (b) 1. *n*-BuLi, THF, −78 °C; 2. 1-benzylpiperidin-4-one, 62%. (c) 1. NaBH_4_, 0.01 equiv. chiral co-complex (*R*,*R*)-**24**, CH_3_OH/CH_2_Cl_2_ 1:1, 0 °C → rt, 30 min; 2. LiAlH_4_, Et_2_O, yield 63%, 95.6%ee. (d) 1. NaBH_4_, 0.01 equiv. chiral co-complex (*S*,*S*)-**24**, CH_3_OH/CH_2_Cl_2_ 1:1, 0 °C → rt, 30 min; 2. LiAlH_4_, Et_2_O, yield 82%, 97.2%ee. (e) TsCl, NEt_3_, DMAP, CH_2_Cl_2_, 17 h, (*R*)-**19** (96.2%ee), (*S*)-**19** (96.4%ee).

However, the separation of enantiomers inevitably leads to 50% waste, since only one of the enantiomers will be of interest for application as a PET tracer in human studies. Therefore, we developed a novel synthesis method with the enantioselective reduction of a double bond as the key step. Thus, the alkyne **22** was prepared by a Sonogashira reaction of 1-bromo-2-iodobenzene (**20**) with the terminal alkyne **21** bearing an orthoester functional group [37]. Bromine–lithium exchange at **22** and subsequent reaction with 1-benzylpiperidin-4-one directly led to the α,β-unsaturated ester **23**. The best method for the enantioselective reduction of the α,β-unsaturated ester **23** appeared to be the reduction with NaBH_4_ in the presence of chiral co-catalysts (*R*,*R*)-**24** and (*S*,*S*)-**24** [38]. The enantiomeric excess was determined after the additional reduction of the ester moiety to a primary alcohol. The reduction of the α,β-unsaturated ester **23** with NaBH_4_ in the presence of (*R*,*R*)-**24** (0.01 equivalents) led to the alcohol (*R*)-**18** in a 63% yield and 95.6%ee. The enantiomeric co-complex (*S*,*S*)-**24** (0.01 equivalents) resulted in an 82% yield of (*S*)-**18** and 97.2%ee [39]. Transformation of the primary alcohols (*R*)-**18** and (*S*)-**18** did not change the enantiomeric excess considerably. As the stereocontrol of the co-catalysts (*R*,*R*)-**24** and (*S*,*S*)-**24** has been thoroughly investigated, the application in the enantioselective synthesis of both fluspidine enantiomers confirmed the assignment of their absolute configuration by CD-spectroscopy (Scheme 3).

In an alternative enantioselective synthesis of both fluspidine enantiomers (*R*)-**2** and (*S*)-**2**, (*Z*)-configured methyl 3-silyloxy-3-(2-bromophenyl)acrylate was enantioselectively reduced with the same co-catalysts (*R*,*R*)-**24**, (*S*,*S*)-**24** and NaBH_4_. However, the establishment of the spirocyclic framework of fluspidine required six additional reaction steps, starting with a Suzuki coupling [40].

Both enantiomers showed very high σ_1_ receptor affinity with *K*_i_ values of 0.57 nM for (*R*)-**2** and 2.3 nM for (*S*)-**2**. Moreover, NMDA receptors with a GluN2B subunit and opioid receptors have been determined to have high selectivity over the σ_2_ subtype.

## 5. In Vitro Characterization of Fluspidine and Its Enantiomers

The lipophilicity of the homologous fluoroalkyl derivatives **1**–**4** was determined by the micro-shake flask method. After the distribution of the compounds between an *n*-octanol layer and MOPS buffer (pH 7.4), the amount of the compound in the buffer layer was determined by mass spectrometry [41,42]. The recorded logD_7.4_ values were compared with the logD_7.2_ values determined by the distribution of the analogous ^18^F-labeled compounds [^18^F]**1**–[^18^F]**4** between *n*-octanol and phosphate-buffered saline, pH 7.2. The logD_7_._2_ values were calculated from the distribution of radioactivity in both layers.

In Table 2, the determined logD_7.4_ and logD_7.2_ values are summarized. As expected, the logD_7.4_ and logD_7.2_ values increased with the increasing length of the fluoroalkyl side chain from 2.80 (**1**) to 3.71 (**4**) and 2.39 (**1**) t0 3.11 (**4**), respectively. The logD_7.4_ and logD_7.2_ values of all four homologs we in a promising range. It should be emphasized that the logD_7.4_ and logD_7.2_ values determined by different methods showed a very good correlation [30,32,33,34].

Plasma protein binding was recorded by HPLC analysis using a stationary phase coated with human serum albumin. The resulting retention times correlate with binding to human serum albumin [42]. The least lipophilic fluoromethyl derivative **1** exhibited the lowest plasma protein binding (84%), which increased with the increasing length of the fluoroalkyl side chain up to 95% for the fluorobutyl derivative **4** (Table 2).

Metabolic stability was initially determined by microsomal assays involving incubation of the homologous fluoroalkyl derivatives **1**–**4** with mouse liver microsomes and NADPH. After an incubation period of 90 min, the amount of remaining test compound was determined by LC-MS [42,43,44]. Under standardized conditions, the metabolic stability decreased with increasing lipophilicity. While the fluoromethyl derivative **1** revealed moderate stability (53% intact after 90 min), the longer homologs **2**, **3** and **4** showed reduced metabolic stability, with only 34%, 33% and 28% of intact parent compounds **2**, **3** and **4** after 90 min, respectively (Table 2).

**Figure 6:**
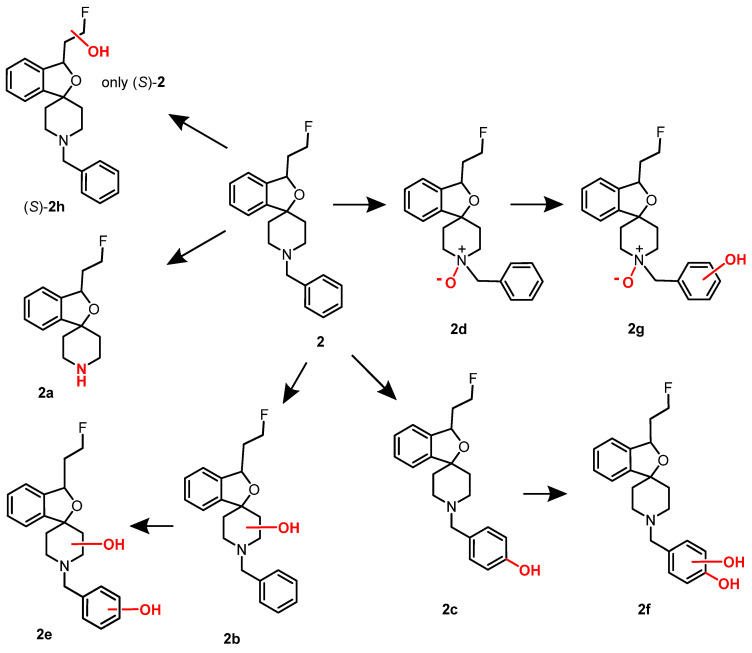
Metabolites formed by incubation of **2**, (*R*)-**2**, and (*S*)-**2** with rat liver microsomes and NADPH [36].

The metabolites formed upon incubation of **2**, (*R*)-**2**, and (*S*)-**2** with rat liver microsomes and NADPH were analyzed by LC-MS and MS^n^ experiments. Figure 6 shows the formed metabolites. The *N*-debenzylated metabolite **2a** and the *p*-hydroxyphenyl metabolite **2c** represent the main metabolites. The structures of both metabolites were confirmed by independent synthesis. Two metabolites bearing OH moieties in the piperidine ring (**2b**) and the fluoroethyl side chain (**2h**) as well as the *N*-oxide **2d** could be detected. Furthermore, three metabolites **2e**–**2g** containing two additional O-atoms were detected. Interestingly, the metabolite **2h** with the additional OH moiety in the fluoroethyl side chain was formed only for the (*S*)-configured enantiomer [36].

## 6. Molecular Interactions of Fluspidine Enantiomers with the σ_1_ Receptor

**Figure 7:**
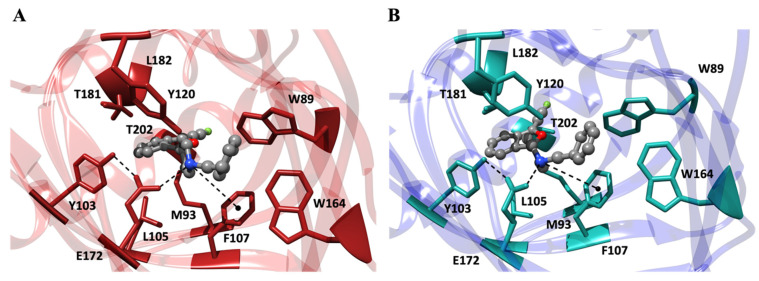
Details of (*R*)-fluspidine (**A**) and (*S*)-fluspidine (**B**) in the binding pocket of the σ_1_ receptor. Compounds are shown as atom-colored sticks-and-balls (C, grey, N, blue, O, red, F, green), while the side chains of the protein residues mainly interacting with the ligands are depicted as colored sticks and labeled. Hydrogen atoms, water molecules, ions, and counterions are omitted for clarity.

Molecular dynamics (MD) simulations were employed to elucidate the interactions between the two fluspidine enantiomers (*R*)-**2** and (*S*)-**2** and the σ_1_ receptor. The X-ray crystal structure 5HK1 [45] of the σ_1_ receptor from the PDB repository was used for the computational studies. The computational investigation primarily focused on evaluating their binding affinity, examining the patterns of interaction, and assessing potential selectivity based on the orientation of key functional groups. For this purpose, both enantiomers (*R*)-**2** and (*S*)-**2** were modeled and docked into the binding site of the σ_1_ receptor, and the relevant free energy of binding (ΔG_bind_) between the protein and the enantiomers was determined using MM/PBSA calculations [46]. As shown in Figure 7A,B, the analysis of the MD trajectories reveals that both (*R*)-fluspidine and (*S*)-fluspidine bind the σ_1_ receptor via the four canonical forms of interactions [9,23,47,48]: (i) a stable salt bridge is established between the positively charged R_3_NH^+^ group of the piperidine ring of the ligand and the negatively charged COO^-^ group of E172. This interaction is facilitated by an optimal arrangement that involves an additional hydrogen bond with Y103. (ii) The aromatic side chain of F107 contributes to the stabilization of the intermolecular complex through a strong π-cation interaction with the aforementioned protonated *N*-atom of the ligands. (iii) The analysis also highlights the presence of a robust network of hydrophobic and π–π interactions between the benzyl moiety of the fluspidine enantiomers and the aromatic groups of σ_1_ receptor residues W89, Y120, and W164. These interactions effectively secure the ligand within the protein binding cavity. (iv) Furthermore, several additional hydrophobic interactions contribute to the stability of the ligand/receptor complex. These interactions mainly involve the 2-benzofuran ring and the side chains of the σ_1_ receptor residues lining the hydrophobic pocket, namely, M93, L105, T181, L182, and T202.

In summary, modeling investigations suggest that both enantiomers (*R*)-**2** and (*S*)-**2** of fluspidine fit well in the binding site of the σ_1_ receptor by establishing similar stabilizing interactions with the receptor. To quantify these interactions, binding free energy calculations were performed. According to these simulations, the affinities of both enantiomers for the σ_1_ receptor are comparable. In fact, the receptor exhibits a marginally higher affinity for the (*R*)-configured enantiomer over the (*S*)-enantiomer, as indicated by the corresponding ΔG_bind_ values of −11.21 ± 0.19 kcal/mol and −10.96 ± 0.21 kcal/mol, respectively.

**Figure 8:**
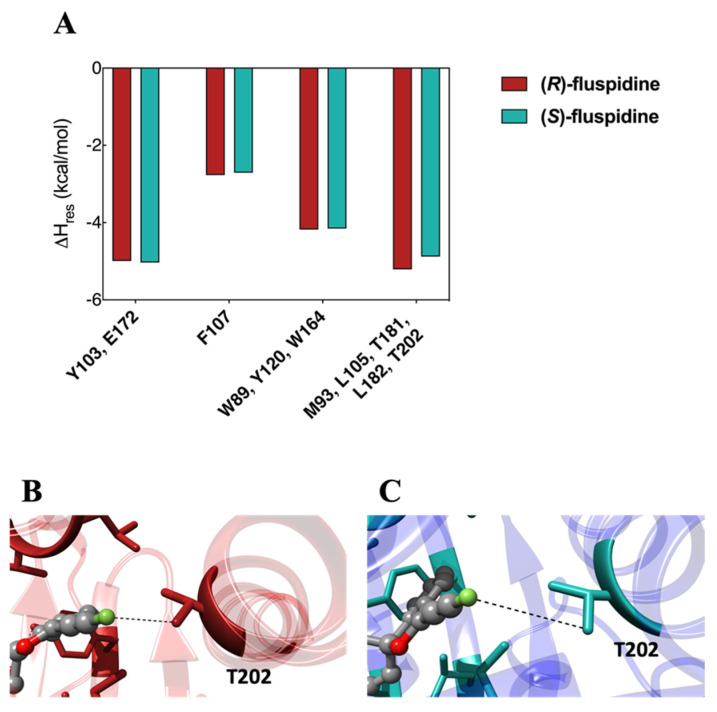
(**A**) Per-residue binding free energy decomposition of the main interacting residues of the σ_1_/(*R*)-fluspidine (firebrick) and σ_1_/(*S*)-fluspidine (light sea green) complexes. Zoomed details of the interaction between the F-atom and T202 hydroxy group in σ_1_/(*R*)-fluspidine (**B**) and σ_1_/(*S*)-fluspidine (**C**) complexes. Compounds are shown as atom-colored sticks-and-balls (C, grey, N, blue, O, red, F, green).

To gain further insight into the receptor binding of the two fluspidine enantiomers, the enthalpic component (ΔH_bind_) of the binding free energy was decomposed into the contributions provided by the protein residues primarily engaged in each ligand binding. (Figure 8A). The results of this analysis indicate that the significant interaction involving E172 has comparable effects on the stabilization of (*R*)-**2** (ΣΔH_res_ = −4.99 kcal/mol) and (*S*)-**2** (ΣΔH_res_ = −5.03 kcal/mol). Additionally, the π-cation interaction with F107 contributes to the stabilization of the (*R*)-**2** and (*S*)-**2** with comparable ΣΔH_res_ values (−2.71 kcal/mol and −2.77 kcal/mol, respectively). Furthermore, the estimated hydrophobic and π-π interactions involving the side chains of residues W89, Y120, and W164 are also almost identical (ΣΔH_res_ = −4.18 kcal/mol for (*R*)-**2** and ΣΔH_res_ = −4.15 kcal/mol for (*S*)-**2**, respectively), supporting the idea that all the interactions described above almost equally contribute to stabilizing (*R*)-**2** and (*S*)-**2** in the binding pocket.

On the other hand, the marginal disparity in binding affinity of the σ_1_ receptor for the two fluspidine enantiomers (*R*)-**2** and (*S*)-**2** can be rationalized by analyzing the stabilizing interactions performed by the ligands in the remaining part of the receptor, that is, the hydrophobic pocket involving residues M93, L105, T181, L182, and T202 (Figure 8B,C), for which the calculated ΣΔH_res_ values are −5.21 kcal/mol and −4.88 kcal/mol for (*R*)-**2** and (*S*)-**2**, respectively.

Beyond these typical hydrophobic interactions, the analysis also revealed that (*R*)-**2** exhibits an additional weak electrostatic contact between the fluoroethyl moiety and the hydroxy group of T202. Accordingly, in the case of (*R*)-**2,** the average dynamic distance (ADD) between these two groups of atoms, as recorded throughout the molecular simulations, is considerably shorter (ADD = 3.98 ± 0.12 Å) compared to that observed for (*S*)-**2** (ADD = 5.15 ± 0.19 Å). In conclusion, the structural and energetic evidence provided in this study provides a molecular-based rationale for the slightly higher binding affinity of the σ_1_ receptor for the (*R*)-configured enantiomer (*R*)-**2** of fluspidine.

## 7. Radiosynthesis

In nuclear medicine, fluorine-18-labeled PET tracers have emerged as powerful tools for non-invasive molecular imaging. The physical and nuclear properties of fluorine-18 (t_½_ = 109.8 min, 97% e^+^ (*β*^+^) decay, e^+^ energy 635 keV) [49] and its availability in cyclotron facilities render fluorine-18 superior to other radionuclides. The broad applicability of fluorine-18 was driven by the development of novel radiofluorination methods [50] and the automate-supported production of PET tracers.

The PET tracers [^18^F]**2**–[^18^F]**4** were prepared by the nucleophilic substitution of the corresponding tosylate precursors with [^18^F]fluoride. For this purpose, the cryptand K_2_._2_._2_ and K_2_CO_3_ were added to the aqueous [^18^F]fluoride solution delivered from the cyclotron, followed by the removal of water by azeotropic drying to obtain “naked” and highly nucleophilic [^18^F]fluoride for the radiolabeling step. The PET tracers [^18^F]**2**–[^18^F]**4** were obtained with 35–51% radiochemical yields, high radiochemical purity and high molar activity [32,33,34] (Table 3). However, the synthesis of the fluoromethyl derivative [^18^F]**1** from the corresponding tosylate required the solvent DMSO at 150 °C instead of CH_3_CN at 85 °C. The solvent DMSO and the higher reaction temperature were necessary due to the branched system (2-benzofuran) in the β-position of the tosyloxy moiety. The branch close to the reaction center inhibited the backside attack of the nucleophile [^18^F]fluoride. Under these reaction conditions, the radiochemical yield, radiochemical purity, and molar activity of [^18^F]**1** were comparable to those of [^18^F]**2**–[^18^F]**4**, while the reaction time was even shorter [30] (Table 3). After the synthesis, PET tracers [^18^F]**1**–[^18^F]**4** were formulated in saline for further investigation and for the first preclinical studies. The total synthesis time was 80–100 min for [^18^F]**1** and 90–120 min for [^18^F]**2**–[^18^F]**4**.

**Scheme 4:**
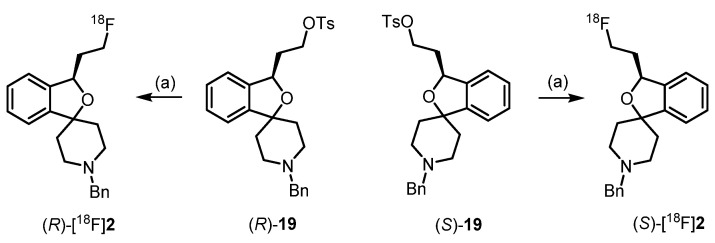
The automated radiosynthesis of enantiomerically pure fluspidine enantiomers (*R*)-[^18^F]**2** and (*S*)-[^18^F]**2**. Reagents and reaction conditions: (a) [^18^F]KF, Kryptofix-2.2.2, 2.5–3 mg tosylate precursor (*R*)-**19** or (*S*)-**19**, CH_3_CN (1 mL), 85 °C, 25 min, radiochemical yield (RCY) 35–45% (N = 10), radiochemical purity (RCP) > 99.6%, molar activity (A_m_) 150–350 GBq/µmol.

Since the PET tracers [^18^F]**2**, (*R*)-[^18^F]**2** and (*S*)-[^18^F]**2** were set for clinical use, automated syntheses were developed using a TRACERlab FX FN synthesis module. The tosylate precursors **19**, (*R*)-**19** and (*S*)-**19** were reacted with [^18^F]fluoride to obtain the PET tracers [^18^F]**2**, (*R*)-[^18^F]**2** and (*S*)-[^18^F]**2** by nucleophilic substitution. After some optimization and adaptation experiments, the PET tracers [^18^F]**2**, (*R*)-[^18^F]**2** and (*S*)-[^18^F]**2** were obtained with high radiochemical yields, high radiochemical purity, high molar activity and short reaction times [51,52] (Scheme 4, Table 4).

## 8. Preclinical In Vivo Studies of Racemic and Enantiomerically Pure [^18^F]Fluspidine [^18^F]2, (*R*)-[^18^F]2 and (*S*)-[^18^F]2

### 8.1. Radiometabolites of rac-[^18^F]2, (R)-[^18^F]2 and (S)-[^18^F]2 In Vivo

**Figure 9:**
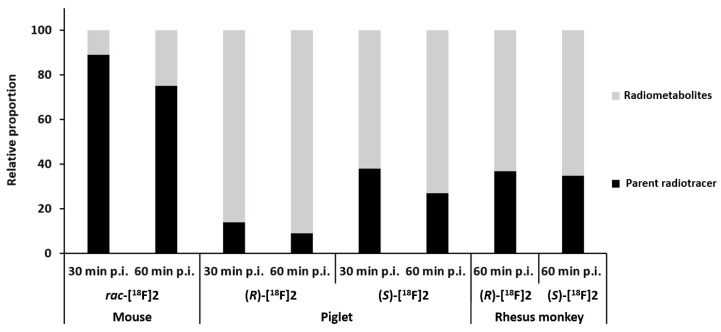
Fractions of unchanged PET tracer and radiometabolites in the plasma of various species determined at 30 and 60 min following intravenous administration of the corresponding radioligand [54,55,56,57,58,59,60,61].

Before detailed preclinical studies were performed using different species, the presence of radiometabolites in the brain was investigated. In the brains of CD-1 mice, 98% of the activity was due to *rac*-[^18^F]**2** 60 min after intravenous administration of the radioligand, indicating the almost complete absence of brain-penetrant radiometabolites [53]. At the same time, the proportion of unchanged PET tracer *rac*-[^18^F]**2** in mouse plasma was still 75%. In piglets, the metabolisms of the two enantiomers (*R*)-[^18^F]**2** and (*S*)-[^18^F]**2** were compared, and plasma samples revealed a higher stability of the (*S*)-configured enantiomer [52], although this was lower than in mice (Figure 9). On the contrary, (*R*)-[^18^F]**2** and (*S*)-[^18^F]**2** showed comparable fractions of unchanged PET tracer in plasma in rhesus monkeys (>30%, 60 min p.i.) [54], which were in the same range as for (*S*)-[^18^F]**2** in piglets.

### 8.2. Organ Distribution in Mice, Piglets and Non-Human Primates

The pharmacokinetic properties of *rac*-[^18^F]**2** were first investigated by ex vivo organ distribution studies carried out in female CD1-mice [32]. Tissues and organs of interest were isolated at different times after the intravenous (i.v.) administration of the radiotracer to measure the radioactivity. Organ activity time data show a rapid, high and slowly increasing uptake of *rac*-[^18^F]**2** in the brain (3.88 and 4.71% of injected dose per g (% ID/g) at 5 and 30 min p.i., respectively), with an activity distribution similar to the immunohistochemical distribution of the σ_1_ receptor in the mouse brain [55]. The rapid uptake in the brain was followed by a slow washout (2.82% ID/g at 120 min p.i.). The significant reduction in activity uptake after blocking the σ_1_ receptor by the previous application of haloperidol confirmed the target specificity of *rac*-[^18^F]**2** in vivo (Table 5).

To evaluate the potential use of the ^18^F-labelled enantiomers (*R*)-[^18^F]**2** and (*S*)-[^18^F]**2** for neuroimaging in a large animal model and to establish a modeling method for the accurate quantification of the σ_1_ receptors in the brain, PET studies were conducted in piglets [52]. These studies showed that both radiotracers (*R*)-[^18^F]**2** and (*S*)-[^18^F]**2** are rapidly taken up throughout the brain with a standardized uptake value (SUV) of about 2 within 3 min after injection. The specific binding of both radiotracers to the σ_1_ receptor in the piglet brain was confirmed by the faster brain washout and the reduced activity observed after the blocking of σ_1_ receptors with SA 4503. Furthermore, the metabolic and brain time–activity curve profiles of both radiotracers indicate the absence of radiometabolites penetrating the blood–brain barrier in the piglets.

However, the differences in the brain uptake kinetics of the two enantiomers in baseline studies indicate that their suitability for diagnostic imaging varies in several pathological situations. Although both enantiomers exhibited similar initial brain uptake, the clearance of (*R*)-[^18^F]**2** was noticeably slower than that of (*S*)-[^18^F]**2** (SUV_120min p_._i_.: ~80% vs. ~50% of SUV_5min p_._i_.). Due to the absence of a reference region with no or very low specific uptake in the pig brain, compartmental modeling and graphical analyses were utilized to analyze the dynamic imaging data to obtain measures for the specific binding of both radiotracers. A full, nonlinear kinetic analysis of the baseline scans using metabolite-corrected plasma input function and two-tissue compartment modeling has revealed whole-brain distribution volumes of *V*_T_ = 13 and 133 mL/g for (*S*)-[^18^F]**2** and (*R*)-[^18^F]**2**, respectively. Furthermore, data obtained from the Logan plot analysis [56] demonstrate a decrease in *V*_T_ values upon SA 4503′s blockade of the σ_1_ receptors (whole brain (*S*)-[^18^F]**2**: −55%, (*R*)-[^18^F]**2**: −89%), confirming the specific binding of both radiotracers.

While the results of the preclinical studies suggest that (*S*)-[^18^F]**2** is appropriate for the quantitative neuroimaging of the σ_1_ receptor and for occupancy evaluations of σ_1_ receptor-targeting drugs [57], the time–activity curves of (*R*)-[^18^F]**2** indicate an apparently irreversible binding to the σ_1_ receptor. This hypothesis was supported by mouse dosimetry studies [58], PET imaging evaluation in non-human primates [54], and in vitro association and dissociation studies [54], demonstrating the extremely slow washout of (*R*)-[^18^F]**2** from the brain in vivo and the negligible dissociation of (*R*)-[^18^F]**2** from the binding site in vitro. However, the distribution pattern of (*R*)-[^18^F]**2** and the much more rapidly cleared (*S*)-[^18^F]**2** in the brain of rhesus monkeys (Table 5) reflect those of other σ_1_ receptor-specific radiotracers in this species. Finally, the rapid brain uptake kinetics, favorable metabolic profile, and high specific signal observed in studies carried out in non-human primates by Baum et al. [54] confirmed the efficacy of (*S*)-[^18^F]**2** in quantifying brain σ_1_ receptor expression levels.

In a mouse model of glioblastoma multiforma, an increased σ_1_ receptor density in the tumor was detected with (*S*)-[^18^F]**2**. An increased σ_1_ receptor density was also observed during an autoradiographic analysis of samples from patients with glioblastoma using (*S*)-[^18^F]**2,** confirming the translational relevance of σ_1_ receptor imaging in oncology [59].

## 9. Human Studies with (*S*)-[^18^F]Fluspidine ((*S*)-[^18^F]2)

### Imaging of σ_1_ Receptors in a Clinical Study (Major Depressive Disorder Patients)

Following preclinical evaluation, the team of Osama Sabri at the Department of Nuclear Medicine of the University of Leipzig, Germany, completed a first-in-man incorporation dosimetry study that initiated successful human PET trials with (*S*)-[^18^F]**2** [60]. The objective of this first clinical proof-of-concept study (DRKS00008321) was to test (*S*)-[^18^F]**2** in patients with major depressive disorder (MDD) compared to healthy subjects, in accordance with evidence for the antidepressant-like effects of agonistic σ_1_ receptor ligands [61].

By kinetic modeling using a 1TCM or 2TCM model, the total volume of distribution (*V*_T_) of (*S*)-[^18^F]**2** can be estimated for all cortical regions from PET 90 min time–activity curves (TACs) with robust and feasible results [62]. Consequently, a PET study with (*S*)-[^18^F]**2** is a suitable method for quantifying σ_1_ receptor availability and related changes in neuropsychiatric diseases due to its high *V*_T_ and short measurement times. In a study on depression conducted in rats, it was found that MDD rats had decreased levels of cardiac σ_1_ receptors [63]. Additionally, the genetic inhibition of the σ_1_ receptor in σ_1_ knock-out mice led to a depressive-like phenotype [17].

Interestingly, in unmedicated patients with acute early-onset MDD, a PET study with (*S*)-[^18^F]**2** revealed an increased availability of σ_1_ receptors in cortico-striato-(para)limbic brain regions compared to healthy volunteers. This increase was strongly correlated with the severity of acute depressive symptoms of MDD and is believed to reflect neuro-adaptive upregulation, which counteracts endoplasmic reticulum (ER) stress [62]. Unfortunately, although the authors of this study suggested additional PET studies with (*S*)-[^18^F]**2** to investigate σ_1_ receptor availability in a larger group of patients with depression in different stages of disease and during treatment, it appears that this study has not been planned or performed, leaving a gap in our understanding of the pathophysiology of the σ_1_ receptor in the context of MDD.

**Figure 10:**
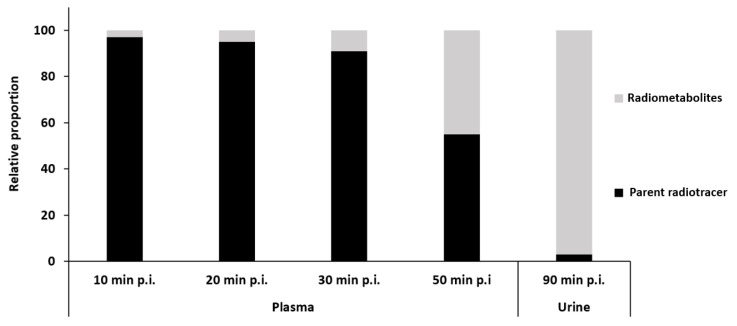
Fractions of unchanged PET tracer and radiometabolites in the plasma and urine of humans determined at different times following the intravenous administration of (*S*)-[^18^F]**2**.

However, during the study mentioned above, the metabolism of (*S*)-[^18^F]**2** was investigated and a high fraction of unchanged PET tracer (*S*)-[^18^F]**2** was found in human plasma (see Figure 10) [62,64].

**Figure 11:**
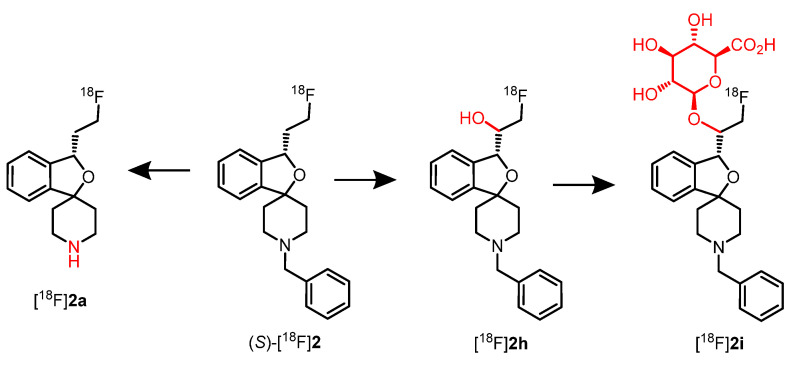
Main radiometabolites formed in humans after administration of (*S*)-[^18^F]**2**.

In contrast, a high fraction of a single radiometabolite, [^18^F]**2i,** and only very small amounts of the unchanged PET tracer (*S*)-[^18^F]**2** were detected in urine. With the aid of in vitro experiments and LC-MS studies, the structures of the main metabolites were elucidated. The glucuronide [^18^F]**2i** represents the main metabolite, which originated from the glucuronidation of the hydroxy metabolite [^18^F]**2h**. The secondary amine [^18^F]**2a** produced by *N*-debenzylation was detected as a minor metabolite of the PET tracer (*S*)-[^18^F]**2** (Figure 11).

**Figure 12:**
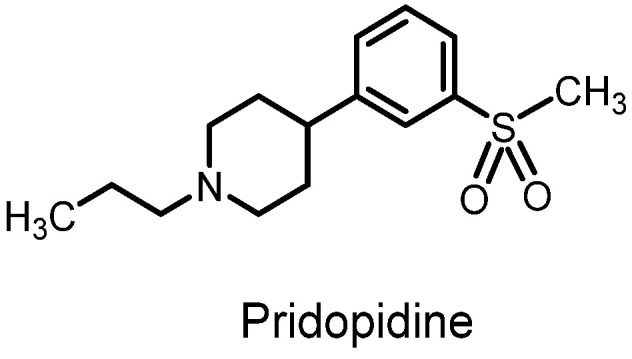
Pridopidine interacting with σ_1_ receptors.

In addition to non-invasively assessing the expression levels of σ_1_ receptors subjected to pathological modifications, supporting the evaluation of σ_1_ receptor as a biomarker for diseases of the CNS, neuroimaging by PET with (*S*)-[^18^F]**2** allows occupancy studies supporting the development of novel therapeutic drugs. The objective of a corresponding study (NCT03019289), undertaken by Osama Sabri’s team, was to demonstrate the target specificity of pridopidine, a drug originally designed for the treatment of CNS pathologies associated with dysfunction of the dopaminergic neurotransmission. Pridopidine (4-[3-(methylsulfonyl)phenyl]-1-propylpiperidine), also known as ACR-16, ASP 2314, FR 310,826 or Huntexil, was originally developed by Arvid Carlsson Research AB as a dopamine stabilizer to reduce hyperactivity and increase behavioral complexity in hypoglutamatergic mice, a model of cognitive deficits in schizophrenia (Figure 12) [65].

The pharmacological properties of this compound made it an interesting candidate for the treatment of a wide range of neurological and psychiatric disorders, such as L-DOPA-induced dyskinesia associated with levodopa treatment in Parkinson’s disease [66,67,68]. Due to neuroprotective effects observed in models of Huntington’s disease (HD) [69], pridopidine was further developed for the treatment of motor symptoms associated with HD. However, different phase 3 studies, initiated by various companies developing pridopidine for clinical applications in HD patients, led to negative results. Therefore, the mechanism of action of pridopidine was reexamined, and a receptor occupancy study in rats indicated the preferential binding of pridopidine to the σ_1_ receptor compared to dopamine D_2_ receptors [69]. In addition, a study in genetically modified mice identified the σ_1_ receptor as a mediator of pridopidine-induced gene expression in the brain, contributing to the neuromodulatory effects of this drug [70]. These findings suggest that the interaction of pridopidine with the σ_1_ receptor in humans should be investigated in more detail.

In (*S*)-[^18^F]**2,** a tool is available that allows the non-invasive evaluation of the occupancy of the σ_1_ receptor by drugs in the human body using PET [71]. The free fraction in blood plasma of (*S*)-[^18^F]**2** available for brain uptake has been determined as *f*_p_ = 0.023. *V*_T_ measurements in healthy volunteers confirmed the results of the preclinical studies performed with (*S*)-[^18^F]**2** in pigs and NHPs discussed above, with values of, e.g., 21 mL/g in the frontal cortex and 19 mL/g in the striatum. The results of the occupancy study using (*S*)-[^18^F]**2** in a PET study indicated the selective binding of pridopidine to the σ_1_ receptor in the brains of healthy volunteers and HD patients at therapeutic doses. On the contrary, only minimal occupancy of dopamine D_2_/D_3_ receptors was found [71].

## 10. Computational Details

The crystal structures of the σ_1_ receptor were obtained from the available PDB file in the Protein Data Bank repository, 5HK1 [45]. All docking experiments were carried out with Autodock 4.2.6/Autodock Tools1.4 [72] on a win64 platform, following a consolidated procedure [9,47,48]. The resulting docked complexes were solvated with explicit TIP3P [73] water, and then the density and the volume of the system were relaxed in the NPT ensemble, maintaining the Berendsen barostat for 20 ns. After this step, 100 ns of unrestrained NVT production simulation was run for each system. According to this computational recipe, ΔG_bind_ values are calculated for equilibrated structures extracted from the corresponding molecular dynamics (MD) by following the MM/PBSA approach [45]. The per residue binding free-energy decomposition was performed using the same MD trajectory of each ligand/σ_1_ complex, with the objective of identifying the key residues involved in the ligand/receptor interaction. This analysis was carried out using the MM/GBSA approach [74], and was based on the same snapshots used in the calculation of the binding free-energy. All simulations were carried out using Amber 21 [75] running on the Marconi100 GPU/CPU supercomputer (CINECA, Bologna, Italy). MarvinSketch was used to draw and display the input chemical structures (Version 23.12.0), ChemAxon Software (http://www.chemaxon.com). All images were created in the UCSF Chimera software (version 1.17.3) [76] and the graphs were produced in GraphPad Prism 8 (GraphPad Software, San Diego, CA, USA, www.graphpad.com).

## 11. Determination of In Vitro Pharmacokinetic Parameters

The pharmacokinetic parameters logD_7.4_ value, plasma protein binding and metabolic stability described in Table 2 were determined according to references [41,42].

## Figures and Tables

**Table 1:** Spirocyclic piperidines with fluoroalkyl side chains.

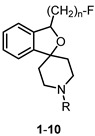					
Compd.	*n*	R	*K*_i_(σ_1_) (nM)	*K*_i_(σ_2_) (nM)	σ_1_/σ_2_ Selectivity
**1**	1	-CH_2_C_6_H_5_	0.74	550	743
**2**	2	-CH_2_C_6_H_5_	0.59	785	1331
**3**	3	-CH_2_C_6_H_5_	1.4	837	598
**4**	4	-CH_2_C_6_H_5_	1.2	489	408
**5**	2	-CH_2_C_6_H_4_-*p*-F	0.57	481	844
**6**	2	-CH_2_C_6_H_11_	0.71	57	80
**7**	2	-CH(CH_3_)C_6_H_5_	1.0	>1 µM	>1000
**8**	2	*n*-butyl	3.9	878	225
**9**	2	*n*-octyl	15	118	7.9
**10**	2	-CH_2_CH=C(CH_3_)_2_	1.5	>1 µM	667

**Table 2:** Pharmacokinetic properties of spirocyclic piperidines **1**–**4** with homologous fluoroalkyl side chain.

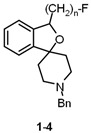						
Compd.	*n*	*K*_i_(σ_1_) (nM)	logD_7.4_^(a)^ Micro Shake Flask	logD_7.2_^(b)^ PET Tracer	Plasma Protein Binding (%)	In Vitro Metabolism—Intact Compd. after 90 min (%)
**1**	1	0.74	2.80 ± 0.04	2.39 ± 0.04	84 ± 0.5	53 ± 3.9
**2**	2	0.59	3.33 ± 0.07	2.57 ± 0.32	88 ± 0.4	34 ± 8.8
**3**	3	1.4	3.60 ± 0.12	2.78 ± 0.06	91 ± 0.4	33 ± 7.9
**4**	4	1.2	3.71 ± 0.14	3.11 ± 0.14	95 ± 0.3	28 ± 4.5

^(a)^ logD_7.4_: *n*-octanol/MOPS buffer pH 7.4, determined by LC-MS. ^(b)^ logD_7.2_: *n*-octanol/phosphate buffered saline pH 7.2, determined by measuring the radioactivity in both layers.

**Table 3:** Radiosynthetic data of the homologous PET tracers [^18^F]**1**–[^18^F]**4** obtained in non-automated synthesis.

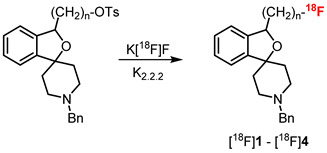						
PET Tracer	Reaction Conditions ^(a)^	RCY_ni_^(b)^ (%)	RCY_fi_^(c)^ (%)	RCP ^(d)^ (%)	A_m_^(e)^ (GBq/µmol)	Time ^(f)^ (min)
[^18^F]**1**	DMSO, 150 °C,	67–86	38–50	>99.1	173–412	80–100
(*n* = 1)	10–15 min	(N = 4)	(N = 3)			
[^18^F]**2**	CH_3_CN, 85 °C,	60–70	35–45	>99.6	150–350	90–120
(*n* = 2)	25 min	(N = 10)	(N = 6)			
[^18^F]**3**	CH_3_CN, 85 °C,	60–70	35–48	>99.5	150–238	90–120
(*n* = 3)	30 min	(N = 11)	(N = 7)			
[^18^F]**4**	CH_3_CN, 83 °C,	60–88	45–51	>98.6	201–528	90–120
(*n* = 4)	20 min	(N = 8)	(N = 7)			

^(a)^ 2.5–3 mg tosylate precursor. ^(b)^ RCY_ni_: radiochemical yield (not isolated). ^(c)^ RCY_fi_: radiochemical yield (final). ^(d)^ RCP: radiochemical purity. ^(e)^ A_m_: molar activity. ^(f)^ Complete synthesis time until final formulation. Parentheses include the number N of experiments.

**Table 4:** Data obtained in the automated radiosynthesis of the racemic and the enantiomerically pure PET tracers[^18^F]**2**, (*R*)-[^18^F]**2** and (*S*)-[^18^F]**2** [51,52].

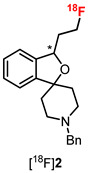					
PET Tracer	Reaction Conditions ^(a)^	RCY_fi_ ^(b)^	RCP ^(c)^	A_m_ ^(d)^ (GBq/µmol)	Time ^(e)^
		(%)	(%)		(min)
[^18^F]**2**	CH_3_CN, 85 °C,	37 ± 8	99.4 ± 0.5	177 ± 52	59 ± 4
	25 min	(N = 9)	(N = 11)	(N = 5)	(N = 8)
(*R*)-[^18^F]**2**	CH_3_CN, 85 °C,	35–45	>99	650–870	70
	15 min				
(*S*)-[^18^F]**2**	CH_3_CN, 85 °C,	35–45	>99	650–870	70
	15 min				

^(a)^ K[^18^F]F, K_2_._2_._2_, 2.5–3 mg tosylate precursor **19**, (*R*)-**19** and (*S*)-**19** using the TRACERlab FX FN synthesis module. ^(b)^ RCY_fi_: radiochemical yield (final). ^(c)^ RCP: radiochemical purity. ^(d)^ A_m_: molar activity. ^(e)^ Complete synthesis time until final formulation. Parentheses include the number of experiments.

**Table 5:** Summary of the distribution pattern of *rac*-[^18^F]**2**, (*S*)-[^18^F]**2**, and (*R*)-[^18^F]**2** binding sites in the brains of mice, piglets and rhesus monkeys.

Species	Parameter (Experimental Details)	Radiotracer	Brain Region (Values)
Mouse [32]	Ratio of activity concentration (target region vs. olfactory bulb at 45 min p.i.; N = 1)	*rac*-[^18^F]**2**	Facial nucleus (4.69), cerebellum (1.75), superficial grey layer of superior colliculus (1.57), cortex (1.45), thalamus (1.24), hippocampus (1.21), striatum (1.11)
Piglet [52]	*V*_T_ in mL/g (Logan plot; baseline conditions; N = 3)	(*S*)-[^18^F]**2**	Midbrain (15.4), colliculi (15.2), cerebellum (14.8), thalamus (14.1), striatum (13.5), hippocampus (13.5), cortex (9.8–13.1)
		(*R*)-[^18^F]**2**	Cerebellum (140), midbrain (132), colliculi (123), thalamus (106), striatum (100), hippocampus (95.2), cortex (67.8–98.4)
Rhesus monkey [54]	*V*_T_ in mL/g (1-Tissue compartment model; baseline conditions; N = 1)	(*S*)-[^18^F]**2**	Cortex (14.6–19.6), hippocampus (16.4), putamen (14.9), amygdala (14.6), caudate (13.5), cerebellum (13.6), caudate (13.5), thalamus (12.2)
		(*R*)-[^18^F]**2**	Cortex (174–291), putamen (199), hippocampus (193), caudate (181), cerebellum (175), amygdala (153), thalamus (128)
